# LACTATE AS PREDICTOR OF MORTALITY IN POLYTRAUMA

**DOI:** 10.1590/S0102-67202015000300004

**Published:** 2015

**Authors:** Andréia Diane FREITAS, Orli FRANZON

**Affiliations:** From the Hospital Regional Homero de Miranda Gomes, Secretaria de Estado da Saúde (Homero Miranda Gomes Regional Hospital, Secretary of State for Health), São José, SC, Brazil

**Keywords:** Lactate, Mortality, Trauma

## Abstract

**Background:**

The lactate is a product of anaerobic metabolism; it can be used as a marker on demand and availability of oxygen. Changes in lactate levels can be effectively used as a marker in resuscitation maneuvers, even in patients with stable vital signs.

**Aim:**

To verify the lactate clearance as a predictor of mortality in trauma patients, in need of intensive care.

**Method:**

A total of 851 patients were admitted in ICU, in which 146 were victims of multiple trauma; due to the exclusion criteria, were included 117.

**Results:**

Patients were 87% male, mean age 32.4 years, motorcycle drivers, Glasgow coma scale between 3-8, affected by cranial trauma, followed by abdominal trauma. Was verified mortality up to 48 h and global mortality, that did not show statistical relationship between lactate clearance and mortality (p=0.928).

**Conclusion:**

There is no correlation between admission lactate or lactate clearance and mortality in patients treated with multiple trauma.

## Abstract (pt)

**Racional:**

O lactato, produto do metabolismo anaeróbio, pode ser utilizado como marcador entre a demanda e disponibilidade do oxigênio. Mudanças nos níveis de lactato podem ser utilizadas como marcador de efetividade nas manobras de ressuscitação, mesmo em pacientes com sinais vitais estáveis.

**Objetivo:**

Verificar o clearance de lactato como preditor da mortalidade entre vítimas de politraumatismo com necessidade de tratamento intensivo.

**Método:**

Um total de 851 pacientes foram admitidos em UTI, sendo que 146 vítimas de politraumatismo, e destes foram incluídos 117 indivíduos, os demais excluídos. As amostras eram homogêneas entre os grupos de sobreviventes e óbitos.

**Resultados:**

Os pacientes eram 87% homens, idade media 32,4 anos, motociclistas, Glasgow entre 3 a 8, acometidos por traumatismo crânio encefálico, seguido de trauma abdominal. Verificada a mortalidade, foi ela dividida em precoce (até e inclusive 48 h) e tardia (após 48 h), sem demonstrar relação estatística entre clearance de lactato e mortalidade (p=0,928).

**Conclusão:**

Não há correlação entre lactato de admissão ou clearance de lactato e mortalidade nos pacientes atendidos com politraumatismo.

## INTRODUCTION

The most common trauma causes of morbidity and mortality are external and responsible about 3,000,000 admissions in the last two years in Brazil 11 . The World Health Organization estimates about 5.8 million annual deaths worldwide by trauma 11 , and 139,648 in Brazil only in 2012 1 . Considering population, the most affected by deaths from external causes are men between 15 to 39 years, productive and contributive 11 . Among causes can be related traffic accidents, falls, drowning, firearms shooting accidents, exposure to smoke, fire and flames, aggression and autoinduced injuries 1 . Victims who do not die may have motor and neurological consequences, either temporary or permanent, with high costs for public allowance, health care and emotional repercussions for families. It is therefore vital early recognition of major injuries and hypovolemic shock 7 10 .

The metabolic response to trauma culminates in inadequate supply of oxygen, hypoxia and anaerobic metabolism, the final product being lactate. It results from the metabolism of pyruvate catalyzed by the enzyme lactate dehydrogenase, found in high concentrations in shock patients 2 5 10 . Victims of trauma, high lactate is proven factor in mortality 2 and may signalize the need for hemoderivatives 9 10 . Checking it in association with blood pressure it is possible to have severe injury indicative 7 10 12 . Some studies have linked lactate >4 mmol/l as a major criterion of severity and chance of survival, ​​rarely found in stable patients even with comorbidities 6 8 10 . Others show that patients with high blood lactate have higher risk of death compared to those with levels within the normal laboratory range 3 4 . The clearance of lactate may represent good parameter to analyze the quality of resuscitation measures in trauma 10 13 and information on prognosis, especially in early mortality. Thus, the lactate can be used as a marker between the demand and availability of oxygen and its level changes can be used as effective marker in resuscitation maneuvers, even in patients with stability in vital signs 7 .

The objective of this study was to analyze the correlation of arterial lactate values ​​on admission and in 6 h clearance with polytrauma mortality and the correlation of the admission lactate with altered vital signs.

## METHODS

The study was submitted for approval by the Research Ethics Committee of the Regional Homero Miranda Gomes Hospital at São José, SC, Brazil before its realization.

It is a retrospective observational cohort, based on multiple trauma patients database admitted in emergency unit and sent to intensive care from April 2013 to July 2014. The variables were: age, gender, mechanism of injury, blood pressure, heart rate, Glasgow coma scale and blood lactate in the first 3 h of hospital admission and between 3 and 9 h afterwards, to calculate the lactate clearance under the following formula: clearance=lactate (lactate admission) - (lactate 6 h) / (lactate admission)x100 7 .

The outcome of each patient was classified in survival or death, with early death if taken less than 48 h after hospital admission, and late if after 48 h.

The sample was separated into two subgroups according to the final outcome, deaths or survivors. To compare the average of the quantitative variables was used the ANOVA test. To compare the groups for the distribution of the relative frequency of qualitative variables was used the two proportions equality test (p<0.05). All data collected were stored and launched in spreadsheets scanned with the SPSS V17, Minitab 16 and Excel Office 2010.

## RESULTS

In the period, the intensive care unit received 851 patients of which 146 were for multiple trauma. Of these 29 were excluded for lack of second lactate collection, resulting the sample in 117 patients.

Respectively among deaths and survivors the data were: 1) there was no significance for age, 37.4 and 33.3 years (p=0.69); 2) predominance of men (87%); 3) systolic blood pressure of 118 mmHg and 114 mmHg (p=0.367) and diastolic 68.7 mmHg and 67.5 mmHg (p=0.287); 4) admission lactate 21.7 mg/dl and 20.6 mg/dl (p=0.168); 5) average length of stay 9.8 and 29.7 days with significance (p<0.001); 6) heart rate of 91.8 and 94.6 bpm (p=0.007) ( Table 1 ).

**TABLE 1:** Results of the analyzed data and lactate

Death (n=32)	Survivors (n=85)	p
Age (average)	37,4 (17,9)	33,3 (13,2)	0,069
Systolic blood pressure (average)	118 (30,3)	114,9 (27,1)	0,367
Diastolic blood pressure (average)	68,7 (16,5)	67,5 (19,8)	0,287
Heart rate (average)	91,8 (16,5)	94,6 (22,4)	0,007
Glasgow (average)	7,4 (4,1)	8,3 (4,1)	0,371
Admission lactate	21,7 (11,7)	20,6 (12,1)	0,168
Admission	9,8 (11)	29,7 (23,5)	< 0,001
Causes of polytrauma
Automotive accident	18,8%	19,0%	0,977
Motorcycle accident	46,9%	26,6%	0,039
Level drop	6,3%	15,2%	0,199
Injury by firearms	12,5%	5,1%	0,170

The higher incidence of trauma mechanism was motorcycle accident,) followed by automotive, level drop, road kill, assault, injury by firearms, stab wound, hanging and blunt abdominal trauma. Among deaths and survivors, the only variable that showed statistical significance was motorcycle accident ( Table 1 ). The predominant injury mechanism was head trauma (51%) followed by abdominal trauma (8.7%) ( Table 2 ).

**TABLE 2:** Outcome and mechanism of injury comparison

Injury	Deaths		Survivors		p
	n	%	n	%
TBI	21	67,7%	36	47,4%	0,055
Abdominal blunt trauma	2	6,5%	9	11,8%	0,405
TBI + thoracic trauma	4	13%	5	6,5%	0,144
TBI + orthopedic trauma	1	3,2%	7	9,2%	0,866
TRM	2	6,5%	3	3,9%	0,578
Trauma complications	1	3,2%	0	0,0%	0,116
TBI + abdominal trauma	0	0,0%	4	5,2%	0,521
Abdominal + thoracic trauma	0	0,0%	4	5,3%	0,193
Thoracic trauma	0	0,0%	3	3,9%	0,521
TBI + vascular trauma	0	0,0%	1	1,3%	0,521
TBI + abdominal + thoracic trauma	0	0,0%	1	1,3%	0,521
Abdominal + orthopedic trauma	0	0,0%	1	1,3%	0,521
Cervical trauma	0	0,0%	1	1,3%	0,521
Vascular trauma	0	0,0%	1	1,3%	0,521

TBI=traumatic brain injury; TRM=spinal cord injury

In assessing the lactate clearance in early deaths, there was no statistical significance (p=0.417) among survivors and deaths ( Table 3 ). When assessing clearance of lactate and late deaths also did not occur relationship (p=0.931). The correlation between lactate clearance and the hospital stay was also analyzed; however, the result was not significant (p=0.862) ( Table 3 ).

**TABLE 3:** Clearance relation between death and hospitalization

CLEARANCE		0 - 29		30 - 59		More than 60		Total		p
		n		%		n	%	n	%	n	%
Deaths 48 h	Survivors	42	95%	45	94%	20	87%	109	93%	0,417
	Deaths	2	5%	3	6%	3	13%	8	7%	
Late deaths	Survivors	30	71%	32	70%	17	74%	79	71%	0,931
	Deaths	12	29%	14	30%	6	26%	32	29%	
Hospitalization	Till 29 days	31	76%	31	70%	17	74%	79	73%	0,862
More than 30 days	10	24%	13	30%	6	26%	29	27%

**FIGURE 1:**
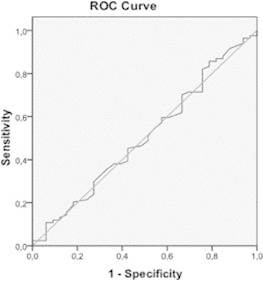
ROC curve of lactate clearance

Lactate clearance among deaths and survivors showed no statistical difference (p=0.920). The ROC curve showed no difference in the clearance between lactate and mortality (area under the curve 0.5, Figure 1 ).

Comparing admission lactate and vital signs, it was found that there was only correlation with systolic blood pressure (18.9%). However, this correlation was classified as very bad ( Table 4 ).

**TABLE 4:** Admission lactate correlation with quantitative variables

Lactate 1	Corr ®	p
Age	-2,0%	0,833
Blood systolic pressure	-18,9%	0,043
Diastolic blood pressure	-15,3%	0,103
Heart rate	15,0%	0,110

When assessing the relationship between deaths and survivors distribution of lactate clearance among low (0 to 29%), moderate (30 to 59%) and high (60% or more), there was no statistical significance among deaths and survivors within the same range ( Table 5 ).

**TABLE 5:** Final outcome and lactate clearance comparison

Clearance	Death		Survivor		p
	n	%	n	%
0 - 29	12	37,5%	30	38,0%	0,963
30 - 59	14	43,8%	32	40,5%	0,753
More than 60	6	18,8%	17	21,5%	0,744

## DISCUSSION

The sample was homogeneous for age and gender. Most patients were men, which coincides with the national polytrauma victims statistics 1 2 and previous studies 5 9 12 . The most affected age group was also in line with other paper^12^, but mortality was higher 12 13 14 .

 Resuscitation in trauma and critically ill patients are challenges; several clinical and laboratory parameters are used to verify the effectiveness of different measurements 15 . The principles in the polytrauma patient care involve recognize and treat bleeding early, limiting the consequences of hypovolemic shock and diagnose traumatic injuries 9 . The ideal marker should be inexpensive, widely available and showing effectiveness of maneuvers in a short time. In an attempt to monitor therapy, lactate can be used 15 .

The trauma response is individual and elderly patients tend to respond distinctively from young people, due to comorbidities, reduced physiological reserve and elasticity of the vascular system, and concomitant use of drugs. It reduces the response to injury and tolerance to aggressive resuscitation measures, while the use of medication can alter the response to shock. In view of these changes, there is need to seek for marker that helps to monitor response acting as predictor of trauma severity 12 .

Lactate is a product of anaerobic metabolism and can be used as a marker of hypoxia in different states of shock. Elevated serum levels on admission of multiple trauma patients is related to higher mortality 3 5 7 15 , higher mortality in patients affected by septic shock 6 and predict blood products need and also may aid in the early detection of severity 3 . Several researchers have established the use of lactate as a diagnostic and prognostic marker of severity and mortality 1 7 9 10 . Levels greater than 4 mmol/l are unusual and are related to systemic inflammatory response and need for treatment in intensive care unit.

In other studies, it was demonstrated pre-hospital lactate as a better predictor of severity and need for surgical treatment in polytrauma in relation to vital signs 5 .

Odom et cols 10 showed great correlation with the initial lactate and mortality. In the present paper such correlation was not observed, even with clearance and mortality in trauma.

Caputo et al 3 demonstrated that vital signs may not be the best predictors of severity in multiple trauma and credited to lactate best positive predictor of mortality. Here, there was no correlation between lactate and blood pressure, lactate and heart rate. In this study, admission lactate showed no correlation with heart rate values ​​and diastolic blood pressure. In addition, those authors demonstrated that elevated lactate as a result of tissue injury and hypoxia was changed even in patients with blood pressure levels within the normal range, since they were young with good hemodynamic compensation after injury. In this study, the heart rate showed to be correlated with mortality; however, the lactate behaved as an independent variable, while vital signs and lactate showed no correlation. 

Hyperlactatemia results from cellular injury and hypoxia, with the ability to demonstrate early cellular suffering, even before the change in vital signs, and assist in screening and pre-hospital treatment 5 . Lactate was used as a predictor in the pre-hospital to need to refer patients to specialist teams in trauma 4 and for early identification of tissue hypoperfusion. High lactate serum levels (>4 mmol/l) also correlated with need for surgical treatment of multiple organ failure and death 4 . In this study, there was no correlation of lactate levels with mortality.

Lactate clearance is reported as a predictor of mortality in patients with stable vital signs or volume loss of less quantities 5 6 12 . In this study, these variables were independent, probably due to limited sample, differing from other authors 4 5 7 10 .

Lefering ET cols 6 demonstrated correlation of serum lactate levels with mortality, with the highest levels present in patients with late mortality, after 48 h of admission. In this study, no correlation was found between lactate and death, either early or late.

There is a strong association of lactate with collagen synthesis and angiogenesis, as an intermediary in the cellular repair process, with relative stability of lactate levels in patients who are hypoxic and then in hyperoxic 8 . They claim that rapidly proliferating cells use glycolysis independent in oxygen levels 8 . Lactate can act in vasodilation. There is evidence pointing to lactic acidosis by glycolysis resulting from the activity of Na + K + ATPase directed to activation of beta adrenergic receptors 8 .

Chana et cols 4 found lower mortality in patients who had greater reduction in their lactate levels, which is a possible way of evaluating the therapeutic instituted in multiple trauma patients.

The relationship between initial high lactate and normal blood pressure levels was observed by other authors 10 and reflects the occult hypoperfusion - groups with high mortality had normal blood pressure levels.

Odom et et al demonstrated that initial lactate is an independent predictor of mortality, and in this study, these variables were not correlated. Furthermore, these authors showed that initial systolic blood pressure and lactate can be predictors of mortality when pressure values ​​are changed. Still, lactate is independent variable of age, Glasgow and injury rates, while in the present study did not detect correlation of lactate with age.

## CONCLUSION

There is no correlation between admission lactate or lactate clearance and mortality in patients treated with multiple trauma.
